# Method for selective ablation of undifferentiated human pluripotent stem cell populations for cell-based therapies

**DOI:** 10.1172/jci.insight.142000

**Published:** 2021-04-08

**Authors:** Tony Chour, Lei Tian, Edward Lau, Dilip Thomas, Ilanit Itzhaki, Olfat Malak, Joe Z. Zhang, Xulei Qin, Mirwais Wardak, Yonggang Liu, Mark Chandy, Katelyn E. Black, Maggie P.Y. Lam, Evgenios Neofytou, Joseph C. Wu

**Affiliations:** 1 Stanford Cardiovascular Institute,; 2 Department of Medicine, Division of Cardiology, and; 3 Department of Radiology, Stanford University School of Medicine, Stanford, California, USA.; 4 Department of Medicine, Division of Cardiology, University of Colorado, Aurora, Colorado, USA.

**Keywords:** Cardiology, Stem cells, Cardiovascular disease, Stem cell transplantation

## Abstract

Human pluripotent stem cells (PSCs), which are composed of embryonic stem cells (ESCs) and induced pluripotent stem cells (iPSCs), provide an opportunity to advance cardiac cell therapy–based clinical trials. However, an important hurdle that must be overcome is the risk of teratoma formation after cell transplantation due to the proliferative capacity of residual undifferentiated PSCs in differentiation batches. To tackle this problem, we propose the use of a minimal noncardiotoxic doxorubicin dose as a purifying agent to selectively target rapidly proliferating stem cells for cell death, which will provide a purer population of terminally differentiated cardiomyocytes before cell transplantation. In this study, we determined an appropriate in vitro doxorubicin dose that (a) eliminates residual undifferentiated stem cells before cell injection to prevent teratoma formation after cell transplantation and (b) does not cause cardiotoxicity in ESC-derived cardiomyocytes (CMs) as demonstrated through contractility analysis, electrophysiology, topoisomerase activity assay, and quantification of reactive oxygen species generation. This study establishes a potentially novel method for tumorigenic-free cell therapy studies aimed at clinical applications of cardiac cell transplantation.

## Graphical abstract

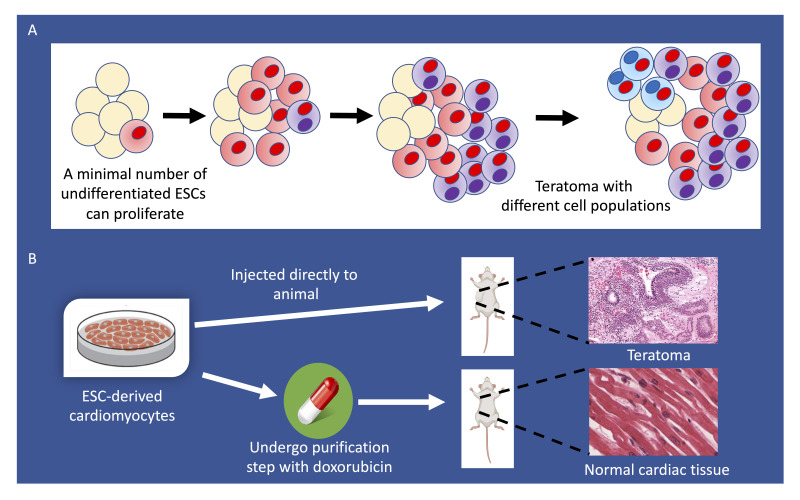

## Introduction

Over the last two decades, rapid advances in pluripotent stem cells (PSCs), which comprise embryonic stem cells (ESCs) and induced pluripotent stem cell (iPSCs), have now allowed regenerative therapy in clinical trials (1, 2). Combined with novel methods to efficiently produce PSC-derived cardiomyocytes (CMs), regenerative therapies to treat heart diseases are now feasible (3). Most regenerative therapies targeting cardiovascular disease involve using stem cell–based therapies to replace injured myocardium and repair injured cardiac tissue to restore ventricular function (4). In the case of cell therapy for patients with heart disease, transplanted cells are intended to engraft onto the endogenous myocardium to restore or enhance cardiac function. However, engraftment is complicated by factors such as constant heartbeat, inflammation, cell leakage, ischemic environment, and hypoxic conditions (5). Currently, the majority of injected cells will neither engraft nor survive following implantation. Injecting a higher cell dosage to compensate can proportionally increase the risk of residual undifferentiated PSCs, and hence, the risk of unintended teratoma formation (6, 7).

Recent studies have highlighted the tumorigenicity risk of stem cell–derived products linked to residual undifferentiated populations of PSCs that persist in the final product (8). PSCs that remain dormant and refractory to differentiation in cultures can be hazardous when transplanted into humans as they can lead to tumor formation. Furthermore, the genetic or epigenetic alterations in proteins or microenvironment changes that occur during the iPSC reprogramming stage might increase the risk of tumor formation after their therapeutic transfer. This is especially true in the field of cardiac stem cell therapy. Inadequately differentiated cell populations have been indicated as the primary contributor to tumorigenic risk in the myocardium, thereby highlighting the need to optimize PSC-CM purification efficiency (9). Wang et al. demonstrated that 7 of 7 animals injected with minimal hPSC population (0.03% of entire PSC-CM batch) developed teratomas only 10 weeks after cell transplantation (10). This complication is a major barrier to clinical trials involving iPSC-CMs and ESC-CMs, which have led to recent developments to try to establish sensitive methods to detect rare tumorigenic stem cells (as low as 0.0005% within cell populations) in PSC-CM populations to promote clinical translation (10).

Researchers have also sought to eliminate the risk of tumorigenesis through optimizing pluripotency reprogramming methods, the use of vitamin C to block pluripotency reprogramming, or drugs that target the epigenetic machinery involved in reprogramming (11). While these methods may prove to be valuable, they currently target the reprogramming aspect of PSCs. Here, we seek to target the proliferative properties of PSCs. Other attempts to target the proliferative properties of PSCs include the introduction of Ki67 promoter–driven thymidine kinase and ganciclovir to eliminate proliferative neural precursors in PSC-based therapy (12). However, one drawback of this approach is the use of viral constructs to introduce this suicide gene platform, which may have off-target effects. Furthermore, ganciclovir is itself considered a potential carcinogen that may result in downstream unintended consequences of cellular proliferation. Nelson et al. have highlighted the heightened susceptibility of embryonic and pluripotent stem cell DNA damage relative to somatic cell types that results in an apoptotic response, which we leverage in our approach to eliminate residual stem cell populations (13).

In this study, we show that low-dose doxorubicin is an effective and safe method to increase cardiomyocyte purity that removes potential proliferative stem cells from terminally differentiated ESC-CMs. Doxorubicin is a proven chemotherapeutic agent for patients with cancer that has been commonly prescribed since the 1960s (14). Doxorubicin belongs to the class of anthracyclines and is administered intravenously in combination with other therapies to treat a range of cancers, including breast cancer, ovarian cancer, and peritoneal carcinomatosis (15, 16). Doxorubicin inhibits topoisomerse II, which in turn results in inhibition of cancer cell growth and proliferation (17). As the target of doxorubicin, topoisomerase II exists as 2 isoforms: topoisomerase IIα and IIβ. Topoisomerase IIα is exclusive to highly proliferative cell types (i.e., cancerous cells) and topoisomerase IIβ is expressed in almost all cell types (i.e., noncancerous cells). Doxorubicin does not discriminate between the two isoforms. Doxorubicin inhibits the activity of both topoisomerase IIα and topoisomerase IIβ (18). After doxorubicin binds to topoisomerase IIβ, cellular apoptosis pathways are activated and hydrogen peroxide is generated that interferes with cellular stability (19). As a result of reactive oxygen species (ROS) accumulation, NADH dehydrogenase complexes within the cardiomyocyte mitochondria are reduced and superoxide anions are generated that lead to mitochondrial electron transport chain dysfunction and DNA damage (19). Activation of p38 MAPK as a result of an elevated doxorubicin dose has also been cited as a contributor to cardiac apoptosis (20). Therefore, it is crucial to find the optimal doxorubicin dosage that prevents cell proliferation of residual undifferentiated stem cells while being noncardiotoxic toward more terminally differentiated PSC-CMs.

## Results

### Doxorubicin induces cell death and apoptosis at lower doses in ESCs than ESC-CMs.

To investigate the appropriate doxorubicin dosage for preventing cell proliferation without causing adverse side effects, we differentiated an H7 human ESC line expressing tomato luciferase into spontaneously beating cardiomyocytes (ESC-CMs). We chose this ESC line expressing tomato luciferase to track the cell fate with bioluminescence imaging upon subcutaneous injection in NOD/SCID mice. Based on fluorescence-activated cell sorting (FACS) analysis of cells stained for cardiac markers troponin T (cTnT) (13-11, Thermo Scientific, MA5-12960) and α-actinin (H-300, Santa Cruz Biotechnology, sc-15336), we determined that cardiac cell purity was 80%–90%, as is typical with our differentiation protocol (21) (data not shown). These cells were used in all subsequent experiments. To determine the dose-dependent effect of doxorubicin on both ESCs and ECS-CMs, we performed viability assays on cells treated with 0.01, 0.05, 0.1, 0.5, or 1 μmol/L concentration of doxorubicin (Supplemental Figure 1A; supplemental material available online with this article; https://doi.org/10.1172/jci.insight.142000DS1). Approximately 90% cell death was observed in ESCs treated with minimal dose doxorubicin (0.01 μmol/L) for 48 hours compared with untreated ESCs (*P* < 0.05). In contrast, no cell death was noted in differentiated ESC-CMs treated with doxorubicin (0.01–0.05 μmol/L) for 48 hours (Supplemental Figure 1A). We observed ESC-CM death only with the higher dose doxorubicin treatment (0.1–1 μmol/L) for 48 hours.

To further characterize the cytotoxic effects of doxorubicin and determine whether the cytotoxic effects are selective toward undifferentiated ESCs and not differentiated ESC-CMs, we next analyzed cell death and apoptosis by FACS (Supplemental Figure 1B and quantified in Supplemental Figure 1C). We treated cells with doxorubicin for 12 and 24 hours to determine the time frame in which apoptosis and cell death are induced. We assessed ESCs and ESC-CMs treated with 0.01, 0.1, or 1 μmol/L of doxorubicin for up to 24 hours with PI and annexin V staining. We observed a significantly reduced number of live ESCs after 12 and 24 hours of doxorubicin treatment at 0.01, 0.1, and 1 μmol/L compared with untreated ESCs (*P* < 0.05). In contrast, there was no significant difference (*P* < 0.05) in live-cell or apoptotic populations detected in untreated and doxorubicin-treated ESC-CMs at 0.01 μmol/L doxorubicin at 12 and 24 hours. These results validate our finding that a minimal dose of doxorubicin (0.01 μmol/L) is safe to use to purify ESC-CMs, as cells treated with a 10-fold higher doxorubicin concentration dose, likewise, did not exhibit cardiotoxic phenotypes. Furthermore, a 0.01 μmol/L dose of doxorubicin is much lower than the peak concentration of doxorubicin in the plasma of patients given this drug intravenously (5 μmol/L), pointing toward potential future acceptance of this method in the clinical setting (22).

### Differentiated cells are less susceptible to cell death induced by minimal doxorubicin dose.

Next, we determined whether our minimal doxorubicin dose induces cell death in other stem cell–derived products. We treated human embryonic stem cell–derived endothelial cells (ESC-ECs), human embryonic stem cell–derived hepatocytes (ESC-HEPs), human embryonic stem cell–derived neuronal cells (ESC-NCs), and human induced pluripotent stem cell–derived smooth muscle cells (iPSC–SMCs) with increasing doxorubicin dosages and assessed cell viability after 12 hours (Supplemental Figure 2A). We observed a significant drop in cell viability only at higher doses of 0.1–1.0 μmol/L doxorubicin for ESC-ECs (91% viability upon treatment with 0.1 μmol/L doxorubicin), ESC-NCs (94% viability upon treatment with 0.5 μmol/L doxorubicin), ESC-HEPs (88% viability upon treatment with 0.5 μmol/L doxorubicin), and iPSC–SMCs (86% viability upon treatment with 0.5 μmol/L doxorubicin). Notably, for ESC-ECs, our minimal dose doxorubicin (0.01 μmol/L) did not impair NO generation upon stimulation with insulin, which is required in cellular homeostasis (Supplemental Figure 2B). Thus, 0.01 μmol/L of doxorubicin can induce apoptosis and cell death in undifferentiated PSCs, but not differentiated PSC products. This suggests that our selective ablation approach may be useful not only for cardiac cell therapy, but also in other types of differentiated ESC- or iPSC-based therapies.

### Minimal dose doxorubicin does not affect ESC-CM viability, but selectively eliminates ESCs.

Upon establishing a doxorubicin dose that does not induce cardiotoxicity in ESC-CMs, we next determined the potential physiological impact this dose might have on ESC-CMs. We observed no notable differences in ESC-CM viability and beating under bright field microscopy when treated with a minimal dose of doxorubicin (0.01 μmol/L) for 48 hours, whereas a high level of ESC death was observed (Supplemental Figure 3A). We assessed the physiological characteristics of the control ESC-CMs compared with ESC-CMs treated with 0.01 μmol/L doxorubicin for up to 48 hours, starting on day 9 of differentiation (Supplemental Figure 3B). As previously shown, ESC-CM viability is intact 48 hours after the doxorubicin treatment (Supplemental Figure 1A). We set doxorubicin treatment at 48 hours to match the hours of the routine ESC-CM media change treatment. At day 11 of differentiation, we observed synchronous monolayer beating and we recorded contractility measurements on day 30 to determine the extent, if any, of functional dysregulation of ESC-CMs treated with 0.01 μmol/L of doxorubicin. We observed no significant differences in contraction velocity, relaxation velocity, acceleration, contraction duration, or relaxation duration of ESC-CMs treated with 0.01 μmol/L of doxorubicin when compared with untreated ESC-CMs (Supplemental Figure 3C).

**Figure 1:**
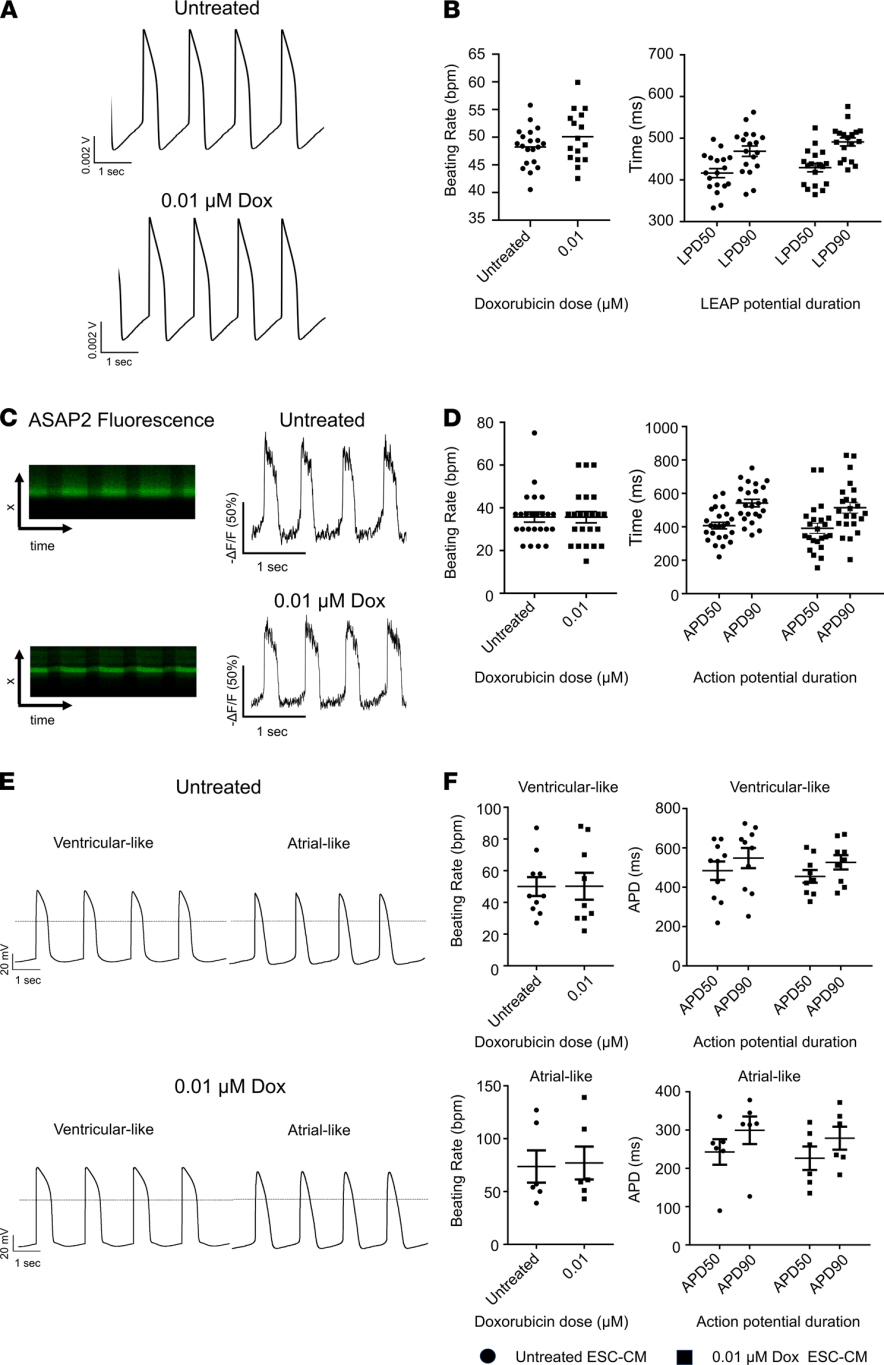
Electrophysiological assessment of ESC-CMs after minimal dose doxorubicin treatment. (**A**) Representative local extracellular action potential (LEAP) tracings recorded at the multicellular monolayer level under control (top) and doxorubicin treatment (bottom) conditions. (**B**) Box-and-whisker plots representing beating rate (left) and LEAP potential duration (LPD) at 50% and 90% repolarization (right; analogous to APD) at the multicellular monolayer level. *n* = 18 per group. (**C**) Representative fluorescent intensities over transmembrane distance and time and action potential tracings at the single-cell level, as measured by the voltage sensor, ASAP2. (**D**) Box-and-whisker plots depicting beating rate (left) and APD at 50% and 90% repolarization (right). *n* = 23 per group. (**E**) Representative AP tracings recorded from patch clamp of single “ventricular-like” control (top left) and doxorubicin**-**treated ESC-CMs (bottom left) and “atrial-like” control (top right) and doxorubicin-treated ESC-CMs (bottom right). (**F**) Scatter plot displaying control and doxorubicin-treated “ventricular-like” (*n* = 10 for untreated, *n* = 9 for 0.01 μmol/L doxorubicin) and “atrial-like” ESC-CMs (*n* = 6 for untreated and 0.01 μmol/L doxorubicin) beating rate (left) and APD (right). Action potential durations at 50% and 90% repolarization (APD50, APD90); and LEAP potential duration at 50% and 90% repolarization (LPD50, LPD90). *n* = 9 per group. Differences between the untreated group and treatment group were not significant. Statistical analysis was performed with a 2-tailed Student’s *t* test comparing viability of untreated cells to cells treated with each doxorubicin dose. *P < 0.05, **P < 0.0001. Data represent mean ± SEM.

Next, we tested the effect of 0.01 μmol/L of doxorubicin for 48 hours on the electrophysiological phenotype of ESC-CMs. The treated ESC-CMs did not show a significant difference in action potential duration (APD) or beating rate at the monolayer multicellular level (Figure 1, A and B). This was assessed by the local extracellular action potential (LEAP) assay of the Maestro Pro multielectrode array (MEA) system. Likewise, there was no significant difference in APD or beating rate at the single-cell level when assessed by the fluorescent voltage sensor ASAP2 (Figure 1, C and D). To further validate the MEA results, we utilized the patch-clamp method to assess both atrial-like and ventricular-like ESC-CM subtypes (Supplemental Table 1). Neither types presented any significant differences in APD or beating rate when compared with their untreated ESC-CM counterparts (Figure 1, E and F). This observation is consistent with previous findings that doxorubicin doses up to 6 μmol/L did not induce changes in beat rate or field potential duration (FPD) in iPSC-CMs (23). Importantly, 48 hours of 0.01 μmol/L doxorubicin treatment did not trigger any form of proarrhythmic events (e.g., early afterdepolarization (EAD), delayed afterdepolarization (DAD), triggered activity) (data not shown). Altogether, the data demonstrate that ESC-CMs treated with minimal dose doxorubicin retain contractility and electrophysiology characteristics similar to those of untreated ESC-CMs.

### Minimal dose doxorubicin does not result in ROS generation or impairment of topoisomerase in ESC-CMs.

Indirect topoisomerase IIβ isozyme targeting has been widely cited as a major mechanism of anthracycline-mediated cardiotoxicity in patients (17). To assess the effect of doxorubicin on topoisomerase, we next determined whether topoisomerase extracted from ESC-CMs retained their capacity to unwind catenated DNA for cleavage upon treatment with doxorubicin. Based on our agarose gel results, we found that topoisomerase II extracted from ESC-CMs treated with minimal dose doxorubicin did not lose its capacity to unwind catenated DNA when compared with topoisomerase II extracted from untreated ESC-CMs (see complete unedited blots in the supplemental material). By contrast, topoisomerase II extracted from ESC-CMs treated with a high doxorubicin dose (1 μmol/L) was unable to produce minicircular DNA bands when added to catenated DNA (Supplemental Figure 4, A and B). Another mechanism of anthracycline-induced cardiotoxicity is the generation of ROS (24). Using a fluorescent dye for oxidation/oxidative stress by ROS (TRITC), we found that minimal dose doxorubicin (0.01 μmol/L) did not result in ROS generation in ESC-CMs, while the higher cardiotoxic doxorubicin dose (1 μmol/L) did result in ROS generation (Supplemental Figure 4, C and D) (25). In contrast, we showed significantly elevated levels of oxidative stress in ESCs treated with a minimal doxorubicin dose compared with untreated ESCs (Supplemental Figure 4, C and D). By analyzing publicly available microarray data (26), we found that significantly enriched pathways of iPSC-CMs treated with highly elevated concentrations of doxorubicin (10 μmol/L) include cellular responses to ROS and DNA damage in a p53-dependent mediate transcriptional activation. It is reassuring that the dose used in this study (0.01 μmol/L) is drastically lower (300-fold) than the dose that resulted in ROS-induced cardiac apoptosis (3 μmol/L) (27). Furthermore, 0.01 μmol/L of doxorubicin treatment resulted in high levels of oxidative stress in ESCs, thereby indicating that 0.01 μmol/L of doxorubicin can be used to selectively eliminate undifferentiated cells through ROS-associated pathways.

### Pretreatment with minimal dose doxorubicin before transplantation prevents teratoma formation.

**Figure 2:**
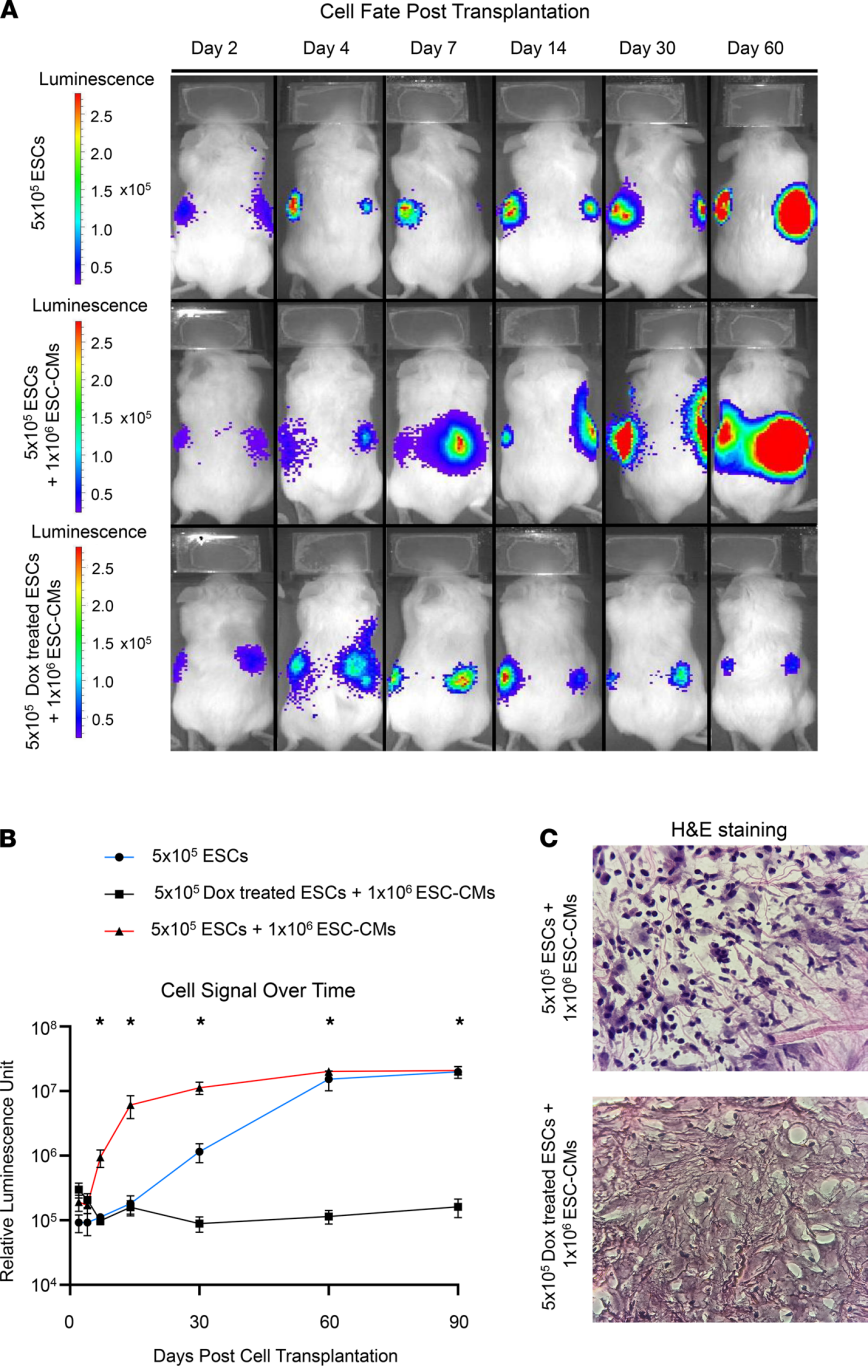
Pretreatment of stem cell products with doxorubicin prevents teratoma formation after in vivo transplantation. (**A**) Representative bioluminescence imaging (BLI) of mice injected with 5 × 10^5^ ESCs (*n* = 5 mice), 5 × 10^5^ ESCs mixed with 1 × 10^6^ ESC-CMs (*n* = 10 mice), or 5 × 10^5^ doxorubicin-treated ESCs mixed with 1 × 10^6^ ESC-CMs (*n* = 10 mice) up to 60 days after cell transplantation. (**B**) Logarithmic plot of bioluminescence signal over a span of 90 days in mice. (**C**) H&E stain of explanted tissue at the site of cell injection in mice injected with either ESC-CMs mixed with ESCs or ESC-CMs mixed with ESCs pretreated with 0.01 μmol/L doxorubicin.

As the aim of our study is to determine the efficacy of minimal dose doxorubicin in eliminating residual undifferentiated ESCs, we spiked our ESC-CMs with additional ESCs to mimic a suboptimal cardiac differentiation batch in which 33% of the cell population consists of noncardiac cells (current monolayer differentiation protocol yield is ~85%–90% ESC-CMs). As such, we mixed 500,000 ESCs that were untreated or treated with 0.01 μmol/L of doxorubicin for 48 hours with 1 million ESC-CMs to obtain the appropriate ratio. Reassuringly, the pretreatment of ESCs with 0.01 μmol/L of doxorubicin for 48 hours before cell transplantation did not result in teratoma formation. We also noted a reduction of the proliferative cell marker Ki-67 in ESC populations after treatment with 0.01 μmol/L of doxorubicin for 48 hours (Supplemental Figure 5). We also characterized cell fate and proliferation after cell transplantation with bioluminescence imaging up to 3 months after cell delivery to monitor cell signals (Figure 2A). Bioluminescence intensity in mice injected with pretreated ESCs remained on the same order of magnitude from the day of injection up to 3 months later, whereas untreated ESCs exhibited a 100-fold higher bioluminescence intensity. These results show that cells pretreated with a minimal doxorubicin dose did not proliferate, whereas untreated ESCs proliferated after cell transplantation (Figure 2B). H&E staining on explanted tissue at the site of cell injection indicates higher cell density in the untreated ESCs mixed with the ESC-CM group when compared with ESCs pretreated with 0.01 μmol/L of doxorubicin and mixed with ESC-CMs (Figure 2C). Therefore, we believe that the minimal in vitro doxorubicin dose has the capacity to prevent residual stem cells in differentiation batches from proliferating and forming teratomas in vivo.

### Evaluating transcriptomic changes in ESCs and ESC-CMs treated with doxorubicin.

**Figure 3:**
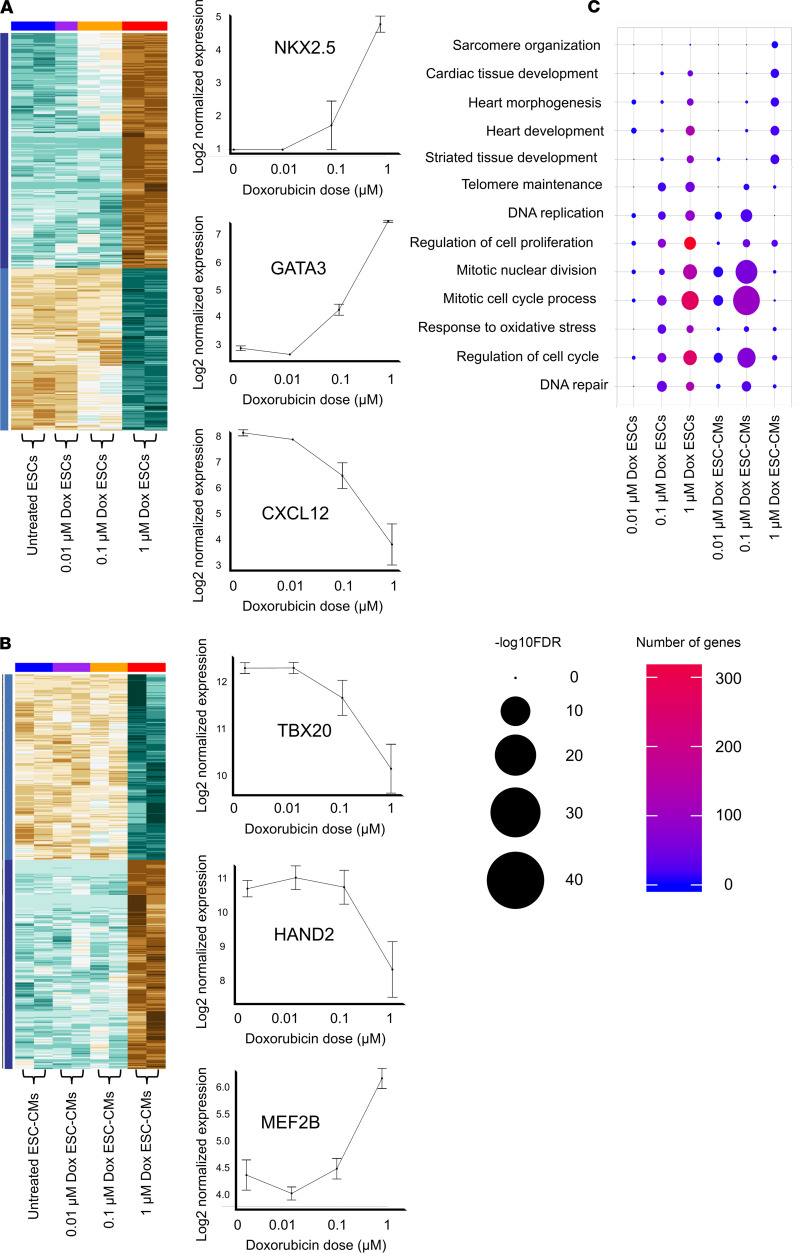
Transcriptomic profiling shows minimal dose doxorubicin does not affect cardiac development pathways in ESC-CMs. (**A**) Heatmap of gene expression levels in ESCs treated with increasing doxorubicin dosages and representative plots of gene expression levels at increasing doxorubicin dosages. (**B**) Heatmap of gene expression levels in ESC-CMs treated with increasing doxorubicin dosages and representative plots of gene expression levels at increasing doxorubicin dosages. (**C**) GoPlot of pathway enrichment and the number of genes involved in each pathway at specific doxorubicin dosages compared with untreated ESCs or ESC-CMs.

We next performed transcriptomic analysis on ESCs and on ESC-CMs treated with 0.01, 0.1, or 1 μmol/L of doxorubicin to characterize the impact of doxorubicin on a molecular level and to determine mechanisms of action in ESC death and ESC-CM cardiotoxicity. Using a principal component analysis (PCA), we depicted cell type–specific transcriptomic differences between ESCs and ESC-CMs (PC1) and visualized transcriptomic patterns for ESCs and ESC-CMs at each individual doxorubicin dose (PC2). We observed the opposite trends in transcriptomic changes between ESCs and ESC-CMs at increasing doxorubicin doses as visualized by PC3, indicating that doxorubicin affects ESC and ESC-CM transcriptome differently (Supplemental Figure 6A). We also compared transcriptomic differences in ESC-CMs relative to ESCs at each specific doxorubicin dose to assess cell type–specific differences (Supplemental Figure 6B). From the transcriptomics data, we identified differentially expressed genes in ESCs and ESC-CMs at each doxorubicin dose and generated heatmaps to visualize dose-specific pathway enrichment (Figure 3, A and B). Side-by-side comparisons of gene expression levels and pathway enrichment of ESCs and ESC-CMs treated with increasing doxorubicin dosage showed slowed regulation of mitotic cell cycle related pathways in ESCs. This indicates a reduction in cell proliferation and upregulation of p53 signaling pathways as well as apoptosis and programmed cell death (Supplemental Figure 7A). In addition, mitochondrial gene expression was profoundly downregulated in ESC-CMs at increasing doxorubicin dosages, suggesting mitochondrial dysregulation (Supplemental Figure 7B).

Interestingly, previous public ChIP-Seq data also corroborate that apoptosis and cell death pathways are induced at the transcriptomic level at a relatively low dose (0.5 μmol/L of doxorubicin in mouse ESCs) (28) (Supplemental Figure 8A). This is also verified by previously published RNA-Seq data showing ROS and mitochondria changes in human iPSC-CMs treated with a high doxorubicin dose (10 μmol/L) (26) (Supplemental Figure 8, B–E). Reassuringly, a comparison of pathway enrichment at each specific doxorubicin dose relative to nontreatment indicates that genes regulating heart development (e.g., sarcomere development, cardiac muscle tissue development, and heart morphogenesis) are not affected at the minimal dose (0.01 μmol/L), but are only affected at a 100-fold higher dose (1 μmol/L) of doxorubicin (Figure 3C).

### Minimal dose doxorubicin does not alter protein expression levels in human ESC-CMs.

**Figure 4:**
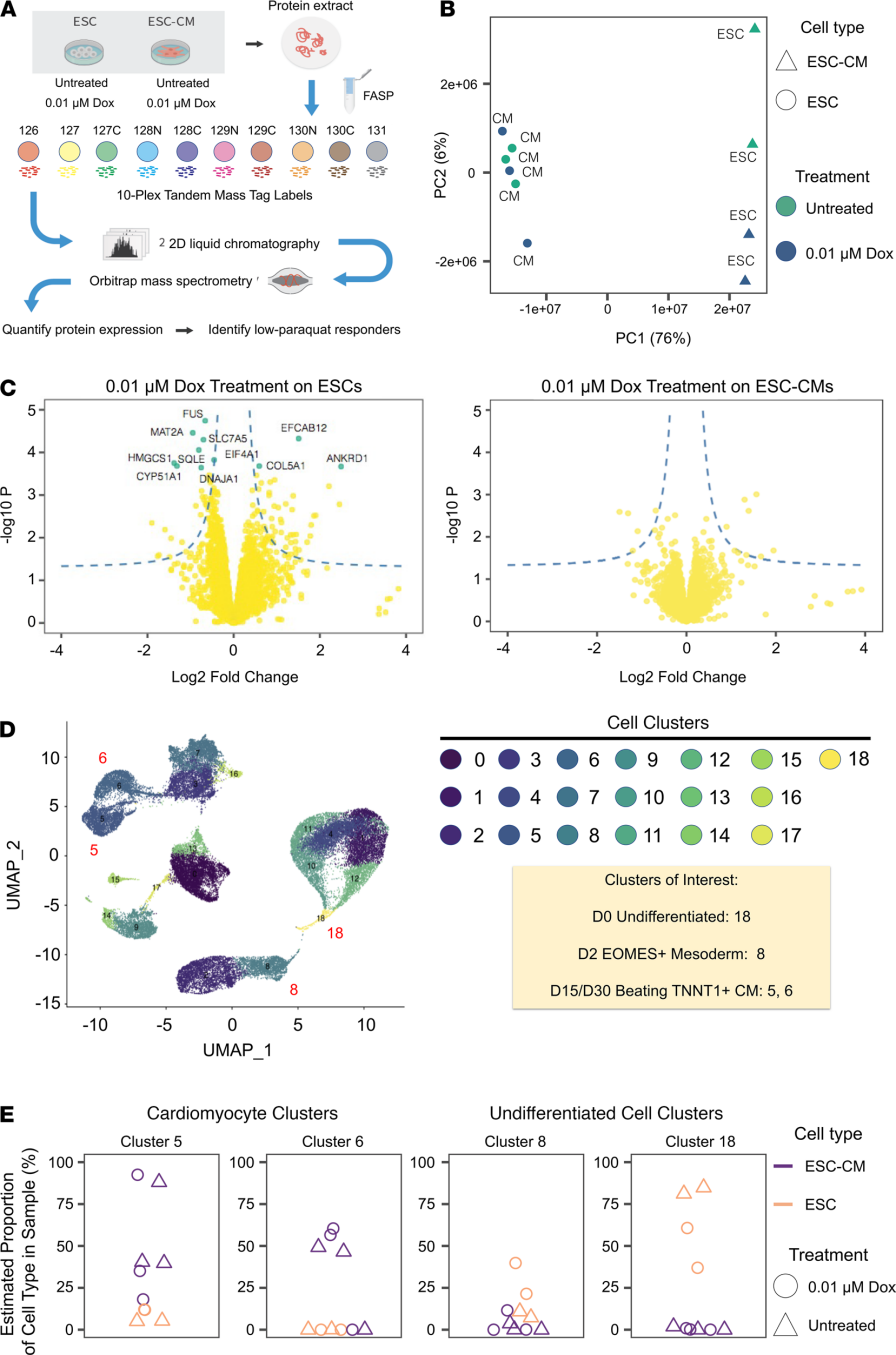
Proteomics analysis shows minimal dose doxorubicin does not alter the proteome profiles of ESC-CMs. (**A**) Workflow diagram in which 50 μg of protein was extracted from control ESCs and ESC-CMs, as well as ESCs and ESC-CMs treated with 0.01 μmol/L of doxorubicin for 48 hours and analyzed using isobaric labeling mass spectrometry. (**B**) PCA plot of protein abundance in ESCs (circles) and ESC-CMs (triangles) that were untreated (green) or treated with 0.01 μmol/L of doxorubicin (blue). (**C**) Volcano plot showing that low-dose doxorubicin caused differential expression of protein coding genes only in ESCs, and not in ESC-CMs, at Benjamini-Hochberg–adjusted *P* (FDR) = 0.01. Dashed line: nominal differential expression at P 0.05 and absolute logFC 0.5; green data points: significant differential expression at 1% FDR. (**D**) UMAP projection of a reprocessed public scRNA-Seq data set (29) at multiple time points of cardiac differentiation corresponding to the gene expression profiles of different cell populations at different stages of development (e.g., cluster 18 shows undifferentiated cells and clusters 5 and 6 correspond to cardiomyocyte-like cells), onto which we mapped the bulk proteomics data to deconvolve the cell type composition of each sample in the mass spectrometry experiment. (**E**) Proportion of each cell type in the bulk proteomics data as estimated with the aid of scRNA-Seq data. The proteomics profile of both the treated and untreated ESC-CM samples in the mass spectrometry experiments corresponded closely with the single-cell gene expression profiles of the cardiomyocyte clusters (5 and 6) with no significant differences in cell composition profiles after doxorubicin treatment. However, doxorubicin treatment in the ESC samples caused a shift in protein abundance that corresponded to a more mesoderm-like gene expression profile, consistent with preferential removal by doxorubicin of cells with high expression of pluripotency markers.

In addition to characterizing the doxorubicin effect on ESCs and ESC-CMs on a transcriptomic level, we were interested in the effect of a minimal dose of doxorubicin on ESCs and ESC-CMs on a proteomic level. Mass spectrometry was performed on ESCs and ESC-CMs treated with our minimal dose doxorubicin (0.01 μmol/L). As expected, a PCA of protein expression profiles showed no significant differences between treated and untreated ESC-CMs but did reveal significant differences between treated and untreated ESCs (Figure 4, A and B). Mass spectrometry analysis shows that 0.01 μmol/L of doxorubicin treatment does not alter protein-coding expression in ESC-CMs, whereas the minimal doxorubicin dose preferentially alters protein-coding expression levels in ESCs (Figure 4C). To further assess how doxorubicin affects the produced ESC-CMs, we deconvolved the bulk proteomic data from each treated or untreated sample into individual cell type compositions with the aid of single-cell RNA sequencing (scRNA-Seq) data. Briefly, we reprocessed a publicly available scRNA-Seq data set of human iPSC-CMs at multiple time points during cardiac differentiation (E-MTAB-6268) (29). Figure 4D shows the uniform manifold approximate and projection (UMAP) of the scRNA-Seq data, including the cell-specific gene expression profiles of iPSC-CMs (clusters 5 and 6) and undifferentiated iPSCs (cluster 8 and 18), onto which we mapped the bulk proteomics data and modeled the bulk proteomics data as a function of cell type–specific gene expression profiles and the proportion of different cell types in the proteomics sample. We used a published algorithm to estimate the proportion of cell types in the proteomics data (Figure 4E) (30). The results indicate that, as expected, protein expression profiles from the ESC-CM samples reflect a near exclusive proportion of iPSC-CM-like cells in the samples. Furthermore, a low-dose doxorubicin treatment did not alter cell composition in the ESC-CM samples. Conversely, protein expression profiles in the ESC samples changed upon doxorubicin treatment, and doxorubicin treatment appeared to preferentially remove a subset of cells, shifting the estimated proportion of cells toward more mesoderm-like expression profiles (Figure 4E and Supplemental Figure 9, A and B). We observed that our minimal doxorubicin dose did not elicit significant differences in protein-coding expression levels in ESC-CMs (clusters 5 and 6) after treatment for 48 hours, whereas significant differences in protein expression levels occurred in ESCs treated for the same amount of time (Benjamini-Hochberg–adjusted *P* [FDR] of 0.01). These data further corroborates the observation that minimal dose doxorubicin preferentially affects undifferentiated ESCs and does not disrupt the proteome integrity of cultured ESC-CMs.

## Discussion

Recent advances in stem cell therapy have shown promise in cardiovascular regenerative medicine. However, the risk of teratoma formation from residual stem cells impedes clinical translation of cell therapy products. Past tumorigenicity studies performed on animal models injected with PSC-CMs have been short term (<3 months) and underpowered. Those studies were unable to detect rare teratoma formations that develop over more extended periods of time after cell transplantation (9). Previous studies have demonstrated the importance of identifying undifferentiated stem cell populations in cell therapy products and proposed novel methods to detect or ablate them (10, 31–35). Once undifferentiated stem cells are detected, practical methods that comply with FDA safety standards must be employed to eliminate these populations without adverse effects on differentiated cell populations (36, 37). For example, radiation therapy has been suggested to reduce stem cell therapy tumorigenicity by using ionizing radiation to cause DNA damage and apoptosis in the stem cells. However, its use is beset by difficulties, including determining the appropriate dose to be administered, side effects on terminally differentiated cells, and potential mutagenic outcomes resulting from DNA damage from ionizing radiation (38–40).

To our knowledge, this is the first study to leverage the antiproliferative properties of doxorubicin, an FDA-approved drug clinically prescribed for cancer therapies, toward purification for cardiac cell therapy applications. We observed the elimination of undifferentiated ESCs in cell cultures and the prevention of teratoma formation in vivo with a minimal in vitro noncardiotoxic doxorubicin dose. In addition, we demonstrated the potential of doxorubicin as a purifying agent against proliferative undifferentiated cells using functional analysis, immunohistochemistry staining, teratoma models, transcriptomic profile, and proteomic analysis. In summary, we have demonstrated the effectiveness and feasibility of a method to reduce stem cell therapy tumorigenicity that promises to advance the translation of stem cell products for cardiovascular clinical applications.

## Methods

### Pretreatment and cell transplantation.

Approximately 5 × 10^5^ H7 ESCs were plated in Essential 8 medium + ROC inhibitor, and 4 hours later, 0.01 μmol/L of doxorubicin was added to each well for our treatment groups. Forty-eight hours after doxorubicin treatment, ESCs were resuspended with 1 × 10^6^ ESC-CMs in Matrigel before subcutaneous flank injections of immunodeficient NOD/SCID mice (*n* = 10). 5 × 10^5^ untreated H7 ESCs were also resuspended with 1 × 10^6^ ESC-CMs in Matrigel before subcutaneous flank injections of immunodeficient NOD/SCID mice (*n* = 10).

### Bioluminescence imaging.

Imaging was performed via IVIS Spectrum at the Stanford Center for Innovation in In-Vivo Imaging. Images were taken for up to 90 days after cell transplantation. Mice were anesthetized via 2% isoflurane and imaged with an exposure time of 30 seconds. One gram of XenoLight D-Luciferin Potassium Salt (PerkinElmer, 122799) was diluted in 23 mL DPBS. 300 μl of this mixture was administered intraperitoneally for bioluminescence imaging via a 28-gauge insulin syringe. A Living Image program was used to analyze the images at different time points. A region of interest (ROI) was drawn over the sites of cell injection. Average radiance was measured in photons/s/cm/sr (steradians).

### Study approval.

All experiments involving animals were performed using protocols approved by the Stanford Administrative Panel on Laboratory Animal Care and Stem Cell Research Oversight committees.

### Statistics.

Statistical analysis was performed with either the 2-tailed Student’s *t* test or 1-way ANOVA. A *P* value of less than 0.05 was considered statistically significant for cell viability, flow cytometry analysis, and contractility assays. All values are expressed as mean ± SEM. Statistical analyses were performed using Microsoft Excel 2013 and PRISM. For transcriptomic analysis, a *P* value of less than 0.01 was considered statistically significant.

## Author contributions

EN and JCW conceived the idea and hypothesis for the study. TC and EN designed the experiments, differentiated ESC-CMs, performed histology, and wrote the manuscript. LT performed analysis of the AmpliSeq, ChIP-Seq, and RNA-Seq data. EL and MPL generated proteomics data and assisted with mass spectrometry analysis. DT performed flow cytometry analysis. II performed MEA experiments. OM performed patch-clamp experiments. JZ performed optical imaging and voltage sensor analysis. XQ, KEB, and MW assisted with manuscript revisions. YL performed cell injections in animal models. MC generated PSC–ECs and performed the nitric oxide release assay. JCW presented experimental concepts, guided with manuscript writing, and provided funding support.

## Supplementary Material

Supplemental data

## Version 1 04/08/2021

Electronic publication

## References

[1] Takahashi K, Yamanaka S (2006). Induction of pluripotent stem cells from mouse embryonic and adult fibroblast cultures by defined factors. Cell.

[2] Menasché P (2020). Cardiac cell therapy: current status, challenges and perspectives. Arch Cardiovasc Dis.

[3] Lian X (2012). Robust cardiomyocyte differentiation from human pluripotent stem cells via temporal modulation of canonical Wnt signaling. Proc Natl Acad Sci U S A.

[4] Müller P (2018). Stem cell therapy in heart diseases - cell types, mechanisms and improvement strategies. Cell Physiol Biochem.

[5] Li X (2016). Improving cell engraftment in cardiac stem cell therapy. Stem Cells Int.

[6] Knoepfler PS (2009). Deconstructing stem cell tumorigenicity: a roadmap to safe regenerative medicine. Stem Cells.

[7] Kooreman NG, Wu JC (2010). Tumorigenicity of pluripotent stem cells: biological insights from molecular imaging. J R Soc Interface.

[8] Sato Y (2019). Tumorigenicity assessment of cell therapy products: the need for global consensus and points to consider. Cytotherapy.

[9] Garbern JC (2021). Pluripotent stem cell-derived cardiomyocytes for treatment of cardiomyopathic damage: current concepts and future directions. Trends Cardiovasc Med.

[10] Wang Z (2020). Ultrasensitive and rapid quantification of rare tumorigenic stem cells in hPSC-derived cardiomyocyte populations. Sci Adv.

[11] Wuputra K (2020). Prevention of tumor risk associated with the reprogramming of human pluripotent stem cells. J Exp Clin Cancer Res.

[12] Tieng V (2016). Elimination of proliferating cells from CNS grafts using a Ki67 promoter-driven thymidine kinase. Mol Ther Methods Clin Dev.

[13] Smith AJ (2012). Apoptotic susceptibility to DNA damage of pluripotent stem cells facilitates pharmacologic purging of teratoma risk. Stem Cells Transl Med.

[14] Mohan P, Rapoport N (2010). Doxorubicin as a molecular nanotheranostic agent: effect of doxorubicin encapsulation in micelles or nanoemulsions on the ultrasound-mediated intracellular delivery and nuclear trafficking. Mol Pharm.

[15] Ottewell PD (2010). Sustained inhibition of tumor growth and prolonged survival following sequential administration of doxorubicin and zoledronic acid in a breast cancer model. Int J Cancer.

[16] Gilliam LA (2011). Doxorubicin causes diaphragm weakness in murine models of cancer chemotherapy. Muscle Nerve.

[17] Nitiss JL (2009). Targeting DNA topoisomerase II in cancer chemotherapy. Nat Rev Cancer.

[18] Marinello J (2018). Anthracyclines as topoisomerase II poisons: from early studies to new perspectives. Int J Mol Sci.

[19] Mizutani H (2005). Mechanism of apoptosis induced by doxorubicin through the generation of hydrogen peroxide. Life Sci.

[20] Kang YJ (2000). Suppression by metallothionein of doxorubicin-induced cardiomyocyte apoptosis through inhibition of p38 mitogen-activated protein kinases. J Biol Chem.

[21] Lian X (2013). Directed cardiomyocyte differentiation from human pluripotent stem cells by modulating Wnt/β-catenin signaling under fully defined conditions. Nat Protoc.

[22] Maillet A (2016). Modeling doxorubicin-Iinduced cardiotoxicity in human pluripotent stem cell derived-cardiomyocytes. Sci Rep.

[23] Louisse J (2017). Assessment of acute and chronic toxicity of doxorubicin in human induced pluripotent stem cell-derived cardiomyocytes. Toxicol In Vitro.

[24] Simůnek T (2009). Anthracycline-induced cardiotoxicity: overview of studies examining the roles of oxidative stress and free cellular iron. Pharmacol Rep.

[25] McGowan JV (2017). Anthracycline chemotherapy and cardiotoxicity. Cardiovasc Drugs Ther.

[26] Burridge PW (2016). Human induced pluripotent stem cell-derived cardiomyocytes recapitulate the predilection of breast cancer patients to doxorubicin-induced cardiotoxicity. Nat Med.

[27] Kim SY (2006). Doxorubicin-induced reactive oxygen species generation and intracellular Ca2+ increase are reciprocally modulated in rat cardiomyocytes. Exp Mol Med.

[28] Li M, An Apela RNA-containing negative feedback loop regulates p53-mediated apoptosis in embryonic stem cells (2015). Cell Stem Cell.

[29] Friedman CE (2018). Single-cell transcriptomic analysis of cardiac differentiation from human PSCs reveals HOPX-dependent cardiomyocyte maturation. Cell Stem Cell.

[30] Wang X (2019). Bulk tissue cell type deconvolution with multi-subject single-cell expression reference. Nat Commun.

[31] Ito E (2019). Tumorigenicity assay essential for facilitating safety studies of hiPSC-derived cardiomyocytes for clinical application. Sci Rep.

[32] Lee AS (2009). Effects of cell number on teratoma formation by human embryonic stem cells. Cell Cycle.

[33] Cao F (2007). Molecular imaging of embryonic stem cell misbehavior and suicide gene ablation. Cloning Stem Cells.

[34] Lan F (2012). Safe genetic modification of cardiac stem cells using a site-specific integration technique. Circulation.

[35] Riegler J (2016). Comparison of magnetic resonance imaging and serum biomarkers for detection of human pluripotent stem cell-derived teratomas. Stem Cell Reports.

[36] Mitsui K (2017). Viral vector-based innovative approaches to directly abolishing tumorigenic pluripotent stem cells for safer regenerative medicine. Mol Ther Methods Clin Dev.

[37] Neofytou E (2015). Hurdles to clinical translation of human induced pluripotent stem cells. J Clin Invest.

[38] Inui S (2017). Irradiation strongly reduces tumorigenesis of human induced pluripotent stem cells. J Radiat Res.

[39] West CM, Barnett GC (2011). Genetics and genomics of radiotherapy toxicity: towards prediction. Genome Med.

[40] Lee AS (2017). Brief report: external beam radiation therapy for the treatment of human pluripotent stem cell-derived teratomas. Stem Cells.

