# Supplementary material for: Method for selective ablation of undifferentiated human pluripotent stem cell populations for cell-based therapies
Source: JCI Insight. 2021 Apr 8;6(7):e142000. doi: 10.1172/jci.insight.142000 (PMC8119193; doi:10.1172/jci.insight.142000)
Supplement: Supplemental data [file jciinsight-6-142000-s233.pdf]

SUPPLEMENTAL MATERIALS

Method for Selective Ablation of Undifferentiated Human Pluripotent Stem Cell Populations for Cell-Based Therapies

Tony Chour[1,2,3], Lei Tian[1,2,3], Edward Lau[1,2], Dilip Thomas[1,2], Ilanit Itzhaki[1,2], Olfat Malak[1,2], Joe Z. Zhang[1,2], Xulei Qin[1,2], Mirwais Wardak[1,3], Yonggang Liu[1,2], Mark Chandy[1,2], Katelyn E Black[1,2], Maggie P.Y. Lam[4], Evgenios Neofytou[1,2], Joseph C. Wu[1,2,3]

[1]Stanford Cardiovascular Institute, [2]Department of Medicine, Division of Cardiology, [3]Department of Radiology, Stanford University School of Medicine, Stanford, CA, [4]Department of Medicine, Division of Cardiology, University of Colorado, Aurora, CO

Correspondence: Joseph C. Wu, MD, PhD, or Evgenios Neofytou MD 265 Campus Drive, Rm 1120B, Stanford, CA94305.

Email: joewu@stanford.edu or neofytou@stanford.edu

SUPPLEMENTARY METHODS

Human ESC culture and maintenance. Human H7 ESC line (Wicell) genetically modified to express tomato luciferase was cultured on Falcon 6-Well Clear Flat Bottom Plates (Corning cat. no: 353046) pre-coated with Corning Matrigel Growth Factor Reduced Basement Membrane Matrix (Fisher Scientific cat no: 356231). 1 mL of 1/200 Matrigel diluted in DMEM/F12 (Gibco/Life Technologies cat. no: 11330-057) was added to each well, and incubated for at least 20 min at 37°C in a New Brunswick Galaxy 170R humidified incubator (Eppendorf) with 5% CO_2 and 5% O_2 prior to addition of cells. The ESCs were replenished with Essential 8™ Medium (ThermoFisher Scientific cat. no: A1517001) daily. The ESCs were dissociated via 0.5 mmol/L UltraPure EDTA, pH 8.0 (Invitrogen cat. no: 15575020) diluted in PBS for passage upon reaching 80-90% confluency. We supplemented 10 μmol/L Y-27632 2HCl (Selleck cat. no: S1049) to dissociate cells prior to plating to prevent cell apoptosis.

Differentiation of embryonic stem cell-derived cardiomyocytes (ESC-CMs). ESC-CM differentiation was performed via a well-defined small molecule Wnt-signaling protocol (1). On day 0, the ESCs were cultured in RPMI 1640 Medium (ThermoFisher Scientific cat. no: 11875093) with B-27™ Supplement minus insulin (ThermoFisher Scientific cat. no: A1895601) supplemented with 6 μmol/L CHIR-99021 (Selleck cat. no: S2924). On day 2, we removed the medium containing CHIR-99021, and we replaced with a basal RPMI 1640 medium supplemented with B27 minus insulin. On day 3, we supplemented 5 μmol/L of IWR-1 (Selleck cat. no: S7086) to the basal RPMI medium minus insulin. On day 5, we replaced the RPMI medium containing IWR with basal medium supplemented with B27 minus insulin. Until day 30, cells were provided with basal medium containing serum-free B-27™ Supplement (50X) (ThermoFisher Scientific cat.

no: 17504044). TrypLE™ Select Enzyme (10X), no phenol red (ThermoFisher Scientific cat. no: A1217701) was added to cardiomyocytes for 10 min at 37°C to dissociate cardiomyocytes into single cells.

Differentiation of embryonic stem cell-derived endothelial cells (ESC-ECs). Human ESCs were differentiated in endothelial cells (ECs) using a monolayer-based protocol previously described (2). From day 0 to day 2, we treated ESCs with 6 µmol/L CHIR99021. From day 2 to day 4, we treated ESCs with 2 µmol/L CHIR to generate the mesoderm. From day 4 to day 12 of differentiation, we treated cells with VEGF, bFGF, and BMP4 in EGM2 media to promote endothelial cell (EC) development. On day 12 of differentiation, the ECs were positively selected for with magnetic activated cell sorting using the bead-conjugated CD 144 antibody.

Differentiation of human induced pluripotent stem cell-derived smooth muscle cells (iPSC-SMCs). Human iPSCs were differentiated into smooth muscle cells (SMCs) using a monolayer-based protocol (3, 4). iPSCs were treated with 6 µmol/L of CHIR99021 from day 0 to day 2, recovered in RPMI+B27-insulin for 24 hr, and treated with 5 µM of IWR-1 (I0161, Sigma) from day 3 to day 5, and then RPMI+B27-insulin for another 24 hr. On day 6, we plated iPSC-derived cardiac progenitor cells at a density of 20,000 cells/cm^2 in Advanced DMEM/F12 medium supplemented with Glutamax (1/100 dilution), ascorbic acid (100 µg/ml), and 5 µM Y-27632 for 24 hr. After one day, cells were maintained in Advanced DMEM/12 medium supplemented with 10 µM SB431542 until confluency. We re-replated pro-epicardial cells in a 1/3-1/4 dilution in advanced DMEM supplemented with PDFG-BB (10 ng/ml) and TGF-beta 1 (2 ng/ml) for 12 days. The medium was changed every other day and cells were passaged upon reaching confluency.

After 12 days of PDGF-BB and TGF-beta 1, we maintained the smooth muscle cells in Lonza SmGM-2 growth medium for 4 weeks.

Differentiation of human embryonic stem cell-derived neuronal cells (ESC-NCs). Human ESCs were treated with Accutase (Innovative Cell Technologies) and plated as dissociated cells in 24 well plates on day -2. The ESCs were plated on Matrigel-(BD Biosciences)-coated coverslips in mTeSR™1. On day -1, lentivirus expressing Ngn2 and EGFP (0.3 µl/well of 24-well plate) was added in fresh mTeSR™1 medium containing polybrene (8 µg/µl, Sigma). On day 0, the culture medium was replaced with N2/DMEM/F12/NEAA (Invitrogen) containing human BDNF (10 µg/l, PeproTech) and human NT-3 (10 µg/l, PeproTech). Doxycycline (2 g/l, Clontech) was added on day 0 to induce TetO gene expression and retained in the medium until the end of the experiment. On day 1, a 24 hr puromycin selection (1 mg/l) period was started. On day 2, we added mouse glial cells in Neurobasal medium supplemented with B27/Glutamax (Invitrogen) containing BDNF and NT3. After day 2, 50% of the medium in each well was exchanged every 2 days. On day 10, we added FBS (2.5%) to the culture medium to support astrocyte viability.

Differentiation of embryonic stem cell-derived hepatocytes (ESC-HEPs). From day 0 to day 3, human ESC-HEPs were differentiated with a monolayer-based protocol that utilizes Activin A 100 ng/ml to induce endoderm differentiation. From day 3 to day 7, FGF 30 ng/ml and BMP2 20 ng/ml were added to specify hepatic cell fate (5).

Cell viability assay. To determine the efficacy of doxorubicin in vitro, we added doxorubicin hydrochloride (Sigma-Aldrich cat. no: 44583-50MG) doses of 0.01, 0.05, 0.1, 0.5, and 1 µmol/L

to H7 human ESCs and differentiated ESC-CMs for 48 hr. Cell viability assays were performed with the CellTiter-Glo 2.0 (Promega cat. no: G9242) kit in which ATP production was measured. Approximately 3,000 ESCs/well were seeded onto a 96-well Matrigel-coated plate (n=8) and 100 μL Essential 8 media was added to each well. Approximately 20,000 ESC-CMs/well were seeded onto a Costar 96-well white plate (Thermo Fisher Scientific cat. no: 07-200-566) (n=8) and 100 μL RPMI + 2% B27 supplement was added to each well. The CellTiter-Glo-2 Reagent was mixed with cells in culture media at a 1:1 ratio. The 96-well plate was incubated at 37°C for 10 min. We used BioTek Gen5 3.03 software to analyze luminescence. Detection was set to the "Absorbance" and read type was set to "Endpoint/Kinetic" level. The integration time was set to 1 sec.

Flow cytometry. For H7 ESCs, $1x10^5$ cells were plated per well on a 12-well plate and allowed to recover for one day prior to doxorubicin treatment (n=3). For ESC-CMs, $3x10^5$ cells were plated per well on a 12-well plate and allowed to recover for one day prior to doxorubicin treatment (n=3). Cells were treated with 0.01, 0.1, and 1 μmol/L of doxorubicin to assess apoptosis and cell death at 12 and 24 hr post-treatment for triplicate samples. Flow cytometry was performed based on the Alexa Fluor® 488 Annexin V/Dead Cell Apoptosis Kit protocol (Thermo Fisher Scientific). Cells were dissociated with 0.5 mmol/L of EDTA in PBS for 5 min and incubated with 2 μL Annexin V antibody in 300 μL 1x Annexin Binding Buffer for 10 min, and 1 μL PI working solution was added to samples immediately preceding flow cytometry. Flow cytometry was performed at the Stanford Shared FACS facility and analyzed with FlowJo software.

Contractility assay. Human ESC-CMs were treated with 0.01 μmol/L of doxorubicin on day 9 of differentiation for 48 hr. On day 20 of differentiation, ESC-CMs were re-plated onto a 6-well plate

(~1x10^6 ESC-CMs/well) and maintained with basal RPMI media (n=15). Contractility was assessed via the Sony SI8000 Cell Motion Imaging System when ESC-CMs reached day 30. Motion detection and analysis were performed with SI8000C software. The motion detection frame interval was set to 4 and the detection threshold was set to 6.

Optical imaging of allosteric sensor for action potentials 2 (ASAP2). Optical imaging of ASAP2 was conducted as previously described (6, 7). Briefly, human ESC-CMs were seeded on a Matrigel-coated 35 mm glass bottom dish with 20 mm micro-well (Cellvis cat. no: D35-10-1.5-N), and infected with ASAP2 lentivirus with the multiplicity of infection (MOI) of five in RPMI-B27 medium. The ASAP2 lentiviral vector was a gift from the laboratory of Michael Lin, and the lentivirus package was conducted by VectorBuilder. The medium containing lentivirus was replaced with fresh RPMI-B27 containing 0.01 μmol/L of doxorubicin 24 hr after infection. Optical imaging of ASAP2 was performed five days after infection. Cells were maintained in Tyrode's solution at 37°C during recording. ASAP2 was excited at 488 nm and emission was collected over 510 nm. Line scan images were acquired on a Zeiss LSM710 confocal microscope (Zeiss) equipped with a 20X objective (NA:0.8). Line scan images were processed to enhance brightness for presentation purposes using ImageJ. Raw imaging data were analyzed using a custom-written MATLAB program. ASAP2 data were presented as -ΔF/F.

Microelectrode array (MEA) recordings. Human ESC-CMs were dissociated enzymatically using Accutase (Sigma). An 8 μl droplet of ESC-CMs in suspension medium (RPMI1640 Medium (1X, Gibco) with B-27 Supplement (50X, Gibco)) was placed over the recording electrode area of each well of the Matrigel-coated 48-well MEA plate and incubated at 37°C in 5% CO_2. After 1 hr

of incubation, an additional volume of 200 μl suspension medium was added to each well of the MEA plate (Axion BioSystems, USA). MEA recordings were performed 14 days after plating. The electrical activity of ESC-CMs was recorded with Maestro Pro (Axion Biosystems) at 37°C using the standard cardiac settings (Axion Biosystems Maestro Axis software). Action potentials (APs) were measured using the Local Extracellular Action Potential (LEAP) assay of the Maestro Pro. Action potential duration was measured as LEAP potential duration (LPD). Data analysis was conducted using the AxIS Navigator analysis tools.

Whole-cell patch-clamp recordings. Human ESC-CM monolayers were enzymatically dispersed (Accutase, Sigma cat. no: SCR005) and attached to Matrigel-coated glass coverslips (Warner, USA). Whole-cell patch-clamp recordings were conducted using an EPC-10 patch-clamp amplifier (HEKA, Germany). 3-4 MΩ glass pipettes were prepared with a micropipette puller (Sutter Instrument, P-97, USA) using thin-wall borosilicate glass (A-M System, USA). Action potentials (APs) were recorded from ESC-CMs superfused with Tyrode solution at 37°C (TC-324B heating system, Warner, USA). The Tyrode solution consisted of NaCl (140 mM), KCl (5.4 mM), $CaCl_2$ (1.8 mM), $MgCl_2$ (1 mM), HEPES (10 mM), and glucose (10 mM); pH was adjusted to 7.4 with NaOH. The pipette solution consisted of KCl (120 mM), $MgCl_2$ (1 mM), Mg-ATP (3 mM), HEPES (10 mM), and EGTA (10 mM); pH was adjusted to 7.2 with KOH. Atrial-like and ventricular-like action potential subtypes were identified by morphology (e.g., "ventricular-like" morphology by a significant plateau phase and "atrial-like" by triangular-shaped action potentials) and the "triangulation ratio" (APD30–40/APD70–80 ratio (RO); Atrial-like: RO ≤1.5; Ventricular-like: RO >1.5) [8-10]. Data were acquired using PatchMaster software (HEKA, Germany) and digitized at 1.0 kHz. Data were analyzed using a custom-written MATLAB program.

Topoisomerase II activity assay. Topoisomerase was extracted from untreated ESC-CMs and ESC-CMs treated with 0.01 or 1 μmol/L of doxorubicin. Topoisomerase activity assay was performed with the Topogen Human Topoisomerase II Assay Kit (Topogen cat. no: TG1001-1A). ImageJ was used to quantify DNA band intensity.

Oxidative stress detection. Human ESCs and ESC-CMs were treated with 0.01, 0.1, or 1 μmol/L of doxorubicin for 48 hr, and oxidative stress was detected and quantified with CellROX Deep Red Reagent (ThermoFisher Scientific cat. no: C10422). Approximately $1x10^4$ ESCs/well and ~$1x10^4$ ESC-CMs/well were seeded onto a Costar 96-well black plate. ImageJ was used to quantify fluorescent intensity.

AmpliSeq. Approximately $1x10^6$ ESCs and ~$1x10^6$ ESC-CMs (day 30) were treated with 0.01, 0.1, or 1 μmol/L of doxorubicin for 24 hr (n=2 per dose). Afterwards, RNA extraction was performed with a RNeasy Mini Kit (Qiagen cat. no: 74104). cDNA was prepared with the Superscript VILO IV Master Mix kit. Library preparation was performed on Ion Chef Library (ThermoFisher Scientific cat. no: 4484177) with the DL8 kit and Ion AmpliSeq™ Transcriptome Human Gene Expression Panel, Chef-ready Kit (cat. no: A31446). The library was prepared with 13 amplification cycles with an anneal and extension time of 16 min according to the Ion AmpliSeq Library Preparation protocol. The barcoded libraries were loaded onto a sequencing chip according to the Ion PI™ Hi-Q ™ Chef Kit protocol. Sequencing was performed on Ion Proton System (ThermoFisher Scientific cat. no: 4476610) following the Ion PI Hi-Q Sequencing 200 Kit protocol.

Human ESC injection for teratoma formation. 8-week old female immunodeficient NOD-SCID mice were anesthetized with 2% isoflurane and subcutaneously injected with $5x10^5$ H7 ESCs (n=5) suspended in 100 µL Matrigel on both left and right flanks as a positive control group. Injection was made via BD Low-Dose U-100 Insulin Syringe 0.5 mL (Fisher Scientific cat. no: 329461). Syringes were kept on ice before injection to prevent the solidification of Matrigel at room temperature.

Teratoma extraction and preservation. Mice were anesthetized and sacrificed prior to teratoma extraction via cervical dislocation. Teratomas were explanted and preserved in paraformaldehyde 20% Solution EM Grade (Electron Microscopy Sciences cat. no: 15713-S) diluted to 4% for 24 hr before transfer into 30% Sucrose (Sigma Aldrich cat. no: 84097-5KG) for 48 hr. Teratomas were flash frozen in 30x22x20 mm Peel-A-Way Embedding Molds (VWR cat. no: 15160-270) containing Tissue-Tek® O.C.T. Compound (VWR cat. no: 4583) via Hexanes (EMD cat. no: HX0290-6) and dry ice solution and stored at -80 °C for histology.

Histology & immunohistochemistry. The flash-frozen teratoma samples were cut in 14 µm sections and stained with H & E Stain Kit (American MasterTech cat. no: KTHNEPT) for cell infiltration. Ki-67 immunofluorescence staining was performed on the 14 µm sections. The slides were submerged in 1x Phosphate Buffered Saline, 10X Solution, Fisher BioReagents™ (Thermo Fisher Scientific cat. no: BP3994) three times for washing (5 min per wash). Sections were submerged in 0.05% Triton X-100 Sigma (cat. no: T8787-100ML) for 1 hr to promote permeability followed by two washes in 1X PBS (2 min per wash). Slides were then submerged in 1% Bovine Serum Albumin (Sigma cat. no: A7906-100G) for 1 hr to block nonspecific binding. 1/100 Anti-

Ki67 antibody (Abcam cat. no: ab15580) dilution was used as the primary antibody to stain the sections. Slides were left at 4°C for 1 hr before the addition of 1/200 Goat anti-rabbit IgG (H+L) Cross-Adsorbed Secondary Antibody (ThermoFisher Scientific cat. no: A-11008). Nuclei were counterstained with 1/1000 DAPI solution (BD Biosciences cat. no: 564907). Slides were mounted with Lab Vision™ PermaFluor™ Aqueous Mounting Medium (ThermoFisher Scientific cat. no: TA-030-FM) prior to visualization.

GOChord and GOBubble plots. Gene Ontology (GO) enrichment analysis was performed on the AmpliSeq dataset in R, and GOChord plots were created to visualize expression levels of genes in enriched pathways (P-value <1%). To follow up on our GOChord data, we looked at the relationship between doxorubicin and p53-mediated apoptosis. We created a bubble plot based on publicly released ChiP-seq data from a study involving p53-mediated damage-induced apoptosis in mouse ESCs treated with 0.5 μmol/L of doxorubicin for 8 hr (11). We compared significant doxorubicin-regulated genes in p53 WT or p53 KO lines that have ≥ 1 p53 binding peaks. The area of each circle is proportional to the number of p53 binding peaks and the color scale for the enrichment of mouse ESCs.

Mass spectrometry sample preparation. Human ESCs and ESC-CMs were either untreated or treated with 0.01 μmol/L of doxorubicin for 48 hr. Afterward, cells were trypsinized and resuspended in 300 μl Thermo M-Per tissue lysis buffer (Thermo) with 1x Halt protease and phosphatase inhibitor (Thermo) on ice for 30 min. Samples were sonicated in the Diagenode BioRuptor Pico (15 sec on, 30 sec off, 10 cycles), and protein concentration was measured using the Pierce bicinchoninic acid assay. A total of 10 samples (triplicate ESC-CM ± doxorubicin,

duplicate ESC ± doxorubicin) were extracted. For each sample, 50 µg of proteins were digested using filter-assisted sample preparation (FASP) on 10-kDa MWCO polyethersulfone filters (Thermo Fisher Scientific). Briefly, the proteins were washed with 8 M urea, then exchanged with triethylammonium bicarbonate (100 mM, 100 µL), reduced with tris(2-carboxyethyl)phosphine (3 µL of 0.5 M, 55°C, 30 min), and alkylated with iodoacetamide (7.5 µL of 375 mM, ambient temperature, 30 min). Proteins were digested on-filter (16 hr, 37°C) with sequencing-grade modified trypsin (50:1, sequencing grade trypsin, Promega). Samples were randomized with a random number generator in R and labeled with tandem mass tag 10-plex stable isotope labels (Thermo Pierce) following the manufacturer's protocol, then quenched with 8 µL of 5% hydroxylamine. The labeled peptides were dried with a SpeedVac evaporator, fractionated using pH-10 reversed-phase columns, then injected for liquid chromatography tandem mass spectrometry analysis on a Q-Exactive HF mass spectrometry at the University of Colorado (M.P.L.).

Mass spectrometry. Liquid chromatography-tandem mass spectrometry was performed on technical duplicate injections of peptides fractionated into 7 fractions using pH-10 reversed-phase spin columns (Thermo Pierce). Second-dimension (pH-2) reversed-phase liquid chromatography was performed with an Easy-nLC 1000 nanoflow ultrahigh-pressure liquid chromatography (UPLC) system on an EasySpray C18 column (PepMap, 3-µm particle size, 100-Å pore size; 75 µm x 150 mm; Thermo) in 90-min in a pH-2 reversed-phase gradient. High-resolution mass spectrometry was performed on a Q-Exactive HF hybrid Quadrupole-Orbitrap mass spectrometer (Thermo) coupled to the nano-UPLC through an EasySpray interface. A typical survey scan setting on the Q-Exactive HF was 60,000 resolving power in positive polarity in profile mode from 300

to 1,650 m/z, with lock mass, 30-sec dynamic, 20-msec injection time. MS2 scans were acquired at 60,000 resolution on the top 15 ions with monoisotopic peak selection in FT/FT mode. The collected mass spectra were converted to the open-source [.mzML] XML format using ProteoWizard msconvert v.3.0.11392 under the filter "peakPicking vendor" option (12). Protein sequence database search was subsequently performed against Uniprot SwissProt Homo sapiens database (20410 entries, retrieved 2018-11-12) using the SEQUEST algorithm implemented in standalone Comet v.2018.01 rev.2 (13), followed by Percolator (14) in the Crux v.3.1 Macintosh binary distribution (15). In the comet parameters, tryptic and semi-tryptic peptides within a 10-ppm parent mass window surrounding the candidate precursor mass were allowed, as were 2 missed cleavages with static cysteine carboxyamidomethylation (C +57.021464 Da; Unimod accession #4) modification. Tandem mass tag channel intensities in the identified spectra were retrieved with a Python script using the pymzml package (16). Statistical analysis of differential expression was performed using the moderated t-test and empirical Bayes model in limma (v.3.34.3) in R/Bioconductor (v.3.6) (17) using discrete developmental stages as factors. Proteins with limma-adjusted P-value (FDR) ≤ 0.05 in each comparison are considered to show evidence for statistically significant differential regulation. To deconvolve bulk proteomics expression into cell type abundance, we reprocessed single-cell RNA sequencing (scRNA-seq) data of human iPSC differentiation stages (ArrayExpress E-MTAB-6268) (18) using the Seurat v3 package in R to identify cell cluster and cell-specific markers for iPSC, iPSC-CM, and intermediary cell types (19). Cell type composition was calculated using the MuSiC non-negative least square algorithm (20), where the relative abundance of protein in bulk from mass spectrometry data is modeled as a linear combination of the relative abundance of a gene in each cell type from single cell data, the

average total cell size of each cell type form single cell data, and the relative proportion of each cell type in the sample.

Statistical analysis. Statistical analysis was performed with either the Student's t-test or ANOVA, and a P-value < 0.05 was considered statistically significant for cell viability, flow cytometry analysis, contractility assays. All values are expressed as mean ± s.e.m. Statistical analyses were performed using Microsoft Excel 2013 and PRISM. For transcriptomic analysis, a P-value < 0.01 was considered statistically significant.

SUPPLEMENTARY FIGURE LEGEND

Supplemental Figure 1. Characterization of doxorubicin-induced cell death and apoptosis in human ESCs and ESC-CMs. (A) Following 48 hr treatment, the viability of H7 ESCs and ESC-CMs was quantified by a luminescence assay to determine the extent of cell death caused by increasing the doxorubicin dose. N=8 per treatment group. **(B)** Representative flow cytometry analysis plots of Annexin V and PI staining of H7 ESCs and ESC-CMs to quantify the extent of apoptosis or cell death caused by increasing doxorubicin doses over time. **(C)** Quantification of Annexin V and PI staining of ESCs and ESC–CMs, shown in subpanel (B), to analyze the percentage of apoptotic, live, and dead cells resulting from increasing doxorubicin doses at different time points. Asterisks indicate significant differences in live cell populations per doxorubicin dose in ESCs and ESC-CMs compared to untreated ESCs and ESC-CMs, respectively. N=3 per group. Statistical analysis was performed with two-tailed Student's t-test comparing viability of untreated cells to cells treated with each doxorubicin dose. $*P < 0.05$, $**P < 0.0001$.

Supplementary Figure 2. Minimal doxorubicin dose preferentially affects undifferentiated ESCs. (A) Cell viability assay of pluripotent stem cell-derived subtypes: ESC-derived neuronal cells (ESC-NCs), ESC-derived endothelial cells (ESC-ECs), ESC-derived hepatocytes (ESC-HEPs), and iPSC-derived smooth muscle cells (iPSC-SMCs) treated with increasing doses of doxorubicin for 12 hr. N=8 per treatment group. **(B)** Nitric oxide (NO) production levels in ESC-ECs after treatment with 0.01 μM doxorubicin for 48 hr. Differences between the untreated and treatment groups were not significant. N=3 per treatment group. Statistical analysis was performed

with two-tailed Student's t-test comparing viability of untreated cells to cells treated with each doxorubicin dose. *P < 0.05, **P < 0.0001.

Supplemental Figure 3. Contractile function of ESC-CMs is unaffected by minimal doxorubicin dose used to specifically eliminate proliferating ESCs. (A) Representative brightfield images of ESCs and ESC-CMs (day 30 of differentiation) treated with 0.01, 0.1, or 1 μmol/L of doxorubicin for 48 hr. Outlines indicate areas of highly concentrated ESCs, which is reduced at increasing doxorubicin doses. **(B)** Schematic representation of cardiac differentiation from ESCs and in vitro doxorubicin treatment timeline. **(C)** Contractility analysis of treated ESC-CMs (0.01 μmol/L of doxorubicin at day 9 of differentiation for 48 hr) compared to untreated ESC-CMs (n=15). Parameters recorded include contraction velocity, relaxation velocity, acceleration, contract duration, relaxation duration, contraction deformation distance, and relaxation deformation distance. The y-axis of each plot shows the unit for each type of parameter. Differences between the untreated and treatment groups were not significant. N=14 per group. Statistical analysis was performed with two-tailed Student's t-test comparing viability of untreated cells to cells treated with each doxorubicin dose. *P < 0.05, **P < 0.0001.

Supplemental Figure 4. Minimal doxorubicin dose does not cause cardiotoxicity in ESC-CMs. (A) Representative 1% agarose gel with DNA bands cut with topoisomerase II extracted from ESC-CMs treated with increasing doxorubicin dosages. Bands represent quantity of cleaved DNA. Lanes separated by vertical black line were run on the same gel but noncontiguous. **(B)** DNA band intensity relative to the DNA band intensity of untreated ESC-CMs from 1% agarose gel indicating the amount of DNA cleavage corresponding to topoisomerase activity. N=3 per

group. **(C)** Fluorescence staining of reactive oxygen species (ROS) generated as a result of increasing doxorubicin dosages in ESCs and ESC-CMs with DAPI staining for cell nuclei and TRITC staining for ROS generated. **(D)** Fluorescence intensity from TRITC staining for ROS in ESCs and ESC-CMs at increasing doxorubicin dosages. N=10 per group. Statistical analysis was performed with two-tailed Student's t-test comparing viability of untreated cells to cells treated with each doxorubicin dose. *$P < 0.05$, **$P < 0.0001$.

Supplementary Figure 5. Proliferative marker staining in ESCs treated with minimal doxorubicin dose. Ki-67 staining of untreated ESCs or ESCs treated with 0.01 μmol/L of doxorubicin for 48 hr.

Supplementary Figure 6. Analysis of ESC and ESC-CM transcriptomes upon treatment with individual doxorubicin doses. **(A)** Principal component analysis (PCA) plots to visualize transcriptome patterns in ESCs versus ESC-CMs treated with doxorubicin. PC1 depicts cell type-specific differences (x-axis), while PC3 depicts transcriptomic trends observed in ESCs and ESC-CMs due to increasing doxorubicin dose (y-axis). **(B)** Significantly different pathways between untreated or doxorubicin-treated ESCs and ESC-CMs at increasing doxorubicin doses. Enclosed box contains significantly enriched pathways of differentially expressed genes between ESCs and ESC-CMs at each dose, with its corresponding chord GOChord plot. Heat maps show the distinct gene expression shift of the indicated ESCs to ESC-CMs at each dose compared to other indicated doses. The numbers in the Venn diagram refer to the number of significantly upregulated and downregulated genes between ESCs and ESC-CMs for each doxorubicin dose. Transcriptomic

analysis was based on AmpliSeq of ESCs and ESC-CMs (day 30) treated with 0, 0.01, 0.1, or 1 μmol/L of doxorubicin for 24 hr. Statistical tests were performed using the DESeq2 R package.

Supplementary Figure 7. Evaluation of biological changes in ESCs and ESC-CMs as a result of increasing doxorubicin dose. **(A)** Visualization of select pathways in ESCs derived from enriched gene expression levels observed at increasing doxorubicin doses. **(B)** Visualization of select pathways in ESC-CMs derived from enriched gene expression levels observed at increasing doxorubicin doses.

Supplementary Figure 8. Effect of elevated doxorubicin dose on ESCs and iPSC-CMs resulting in cell death and apoptosis. (A) Doxorubicin induces p53-mediated apoptosis and cell death in mouse ESCs. GOBubble plot of doxorubicin-dependent p53-mediated apoptosis and cell death pathways in mouse ESCs. Circle size correlates with the number of p53 binding peaks in doxorubicin-regulated genes. The plot is based on publicly released ChiP-seq data on mouse ESCs treated with 0.5 μmol/L doxorubicin for 8 hr. Gene Ontology analysis for B-E was performed on a public dataset containing RNA-seq data of human iPSC-CMs (day 30) treated with 10 μmol/L doxorubicin for 24 hr to visualize iPSC-CM pathway enrichment at a high doxorubicin dose. **(B)** GOChord plot of enriched genes involved in response to reactive oxygen species. **(C)** GOChord plot of enriched genes involved in heart morphogenesis and mitochondrial changes. **(D)** GOChord plot of enriched genes involved in the p53 degradation pathway. **(E)** GOChord plot of enriched genes involved in cycle cell arrest and replication.

Supplementary Figure 9. Single-cell RNA-sequencing of human iPSC-CMs. To estimate the proportion of pure cell types in the bulk proteomics data, we reprocessed a public scRNA-seq dataset by Friedman et al. containing single-cell gene expression profiles of 44,020 cells during multiple time points of iPSC-CM differentiation [18]. As in Figure 7D, UMAP projection shows that cells at different differentiation stages (undifferentiated cells, mesoderm-like cells, cardiac progenitors, and cardiomyocyte-like cells) clustered together, as expected, based on their single-cell expression profile. **(A)** The cell identity of each cluster is verified using typical markers for undifferentiated cells (POU5F1), mesodermal cells (EOMES), cardiac progenitors (ISL1), cardiomyocytes (TNNI1 and MYL7), and fibroblasts (THY1). **(B)** The inferred cell types mapped closely to known developmental projection during the iPSC-CM differentiation protocol used in the study by Friedman et al. Taken together, these data support our use of scRNA-seq data and bulk proteomics data to deconvolve the cell type composition of untreated and doxorubicin-treated ESC and ESC-CM samples and assess the effect of doxorubicin on the treated cells.

SUPPLEMENTARY TABLE

Supplementary Table 1. Patch clamp acquired “ventricular-like” and “atrial-like” action potential parameters under untreated conditions. Patch-clamp acquired “ventricular-like” and “atrial-like” action potential parameters under 48 hr of 0.01 μmol/L doxorubicin treatment.

SUPPLEMENTARY REFERENCES

1. Lian, X., et al., *Directed cardiomyocyte differentiation from human pluripotent stem cells by modulating Wnt/β-catenin signaling under fully defined conditions.* Nat Protoc, 2013. **8**(1): p. 162-75.
2. Paik, D.T., et al., *Large-sale single-cell RNA-seq reveals molecular signatures of heterogeneous populations of human induced pluripotent stem cell-derived endothelial cells.* Circ Res, 2018. **123**(4): p. 443-450.
3. Cheung, C., et al., *Generation of human vascular smooth muscle subtypes provides insight into embryological origin-dependent disease susceptibility.* Nat Biotechnol, 2012. **30**(2): p. 165-73.
4. Bao, X., et al., *Long-term self-renewing human epicardial cells generated from pluripotent stem cells under defined xeno-free conditions.* Nat Biomed Eng, 2016. **1**.
5. Song, Z., et al., *Efficient generation of hepatocyte-like cells from human induced pluripotent stem cells.* Cell Res, 2009. **19**(11): p. 1233-42.
6. Ma, N., et al., *Determining the pathogenicity of a genomic variant of uncertain significance using CRISPR/Cas9 and human-induced pluripotent stem cells.* Circulation, 2018. **138**(23): p. 2666-2681.
7. Zhang, J.Z., et al., *A Human iPSC double-reporter system enables purification of cardiac lineage subpopulations with distinct function and drug response profiles.* Cell Stem Cell, 2019. **24**(5): p. 802-811.e5.
8. Zhu, R., et al., *Variability of action potentials within and among cardiac cell clusters derived from human embryonic stem cells.* Sci Rep, 2016. **6**: p. 18544.
9. Cordeiro, J.M., et al., *Identification and characterization of a transient outward K+ current in human induced pluripotent stem cell-derived cardiomyocytes.* J Mol Cell Cardiol, 2013. **60**: p. 36-46.
10. Doss, M.X., et al., *Maximum diastolic potential of human induced pluripotent stem cell-derived cardiomyocytes depends critically on I(Kr).* PLoS One, 2012. **7**(7): p. e40288.
11. Li, M., et al., *An Apela RNA-containing negative feedback loop regulates p53-mediated apoptosis in embryonic stem cells.* Cell Stem Cell, 2015. **16**(6): p. 669-83.
12. Adusumilli, R. and P. Mallick, *Data Conversion with ProteoWizard msConvert.* Methods Mol Biol, 2017. **1550**: p. 339-368.
13. Eng, J.K., et al., *A deeper look into Comet--implementation and features.* J Am Soc Mass Spectrom, 2015. **26**(11): p. 1865-74.
14. The, M., et al., *Fast and accurate protein false discovery rates on large-scale proteomics data sets with Percolator 3.0.* J Am Soc Mass Spectrom, 2016. **27**(11): p. 1719-1727.
15. McIlwain, S., et al., *Crux: rapid open source protein tandem mass spectrometry analysis.* J Proteome Res, 2014. **13**(10): p. 4488-91.
16. Kösters, M., et al., *pymzML v2.0: introducing a highly compressed and seekable gzip format.* Bioinformatics, 2018. **34**(14): p. 2513-2514.
17. Ritchie, M.E., et al., *limma powers differential expression analyses for RNA-sequencing and microarray studies.* Nucleic Acids Res, 2015. **43**(7): p. e47.
18. Friedman, C.E., et al., *Single-cell transcriptomic analysis of cardiac differentiation from human PSCs reveals HOPX-dependent cardiomyocyte maturation.* Cell Stem Cell, 2018. **23**(4): p. 586-598.e8.

19. Butler, A., et al., *Integrating single-cell transcriptomic data across different conditions, technologies, and species.* Nat Biotechnol, 2018. **36**(5): p. 411-420.
20. Wang, X., et al., *Bulk tissue cell type deconvolution with multi-subject single-cell expression reference.* Nat Commun, 2019. **10**(1): p. 380.
